# Mortality reduction benefits and intussusception risks of rotavirus vaccination in 135 low-income and middle-income countries: a modelling analysis of current and alternative schedules

**DOI:** 10.1016/S2214-109X(19)30412-7

**Published:** 2019-11-01

**Authors:** Andrew Clark, Jacqueline Tate, Umesh Parashar, Mark Jit, Mateusz Hasso-Agopsowicz, Nicholas Henschke, Benjamin Lopman, Kevin Van Zandvoort, Clint Pecenka, Paul Fine, Colin Sanderson

**Affiliations:** Department of Health Services Research and Policy, (A Clark PhD, C Sanderson PhD), Department of Infectious Disease Epidemiology (M Jit PhD, P Fine PhD, K Van Zandvoort MSc), and Department of Immunology and Infection (M Hasso-Agopsowicz PhD), London School of Hygiene and Tropical Medicine, London, UK; Division of Viral Diseases, Centers for Disease Control and Prevention, Atlanta, GA, USA (J Tate PhD, U Parashar MD); Modelling and Economics Unit, Public Health England, London, UK (M Jit); World Health Organization, Geneva, Switzerland (M Hasso-Agopsowicz); Cochrane Response, London, UK (N Henschke PhD); Faculty of Epidemiology, and Faculty of Environmental Health, Emory University, Atlanta, GA, USA (B Lopman, PhD); and PATH, Seattle, WA, USA (C Pecenka PhD)

## Abstract

**Background:**

Infant rotavirus vaccines have led to substantial reductions in hospital admissions and deaths due to gastroenteritis, but some studies have reported an elevated risk of intussusception, a rare bowel disorder. This analysis aimed to provide evidence on the potential mortality reduction benefits and intussusception risks of current rotavirus vaccination schedules, and to explore whether alternative schedules could have advantages.

**Methods:**

All 135 low-income and middle-income countries, defined by gross national income per capita of less than US$12 236 in the 2018 fiscal year, were included in the model. Mortality reduction benefits and intussusception risks of rotavirus vaccination were modelled by use of an Excel-based static cohort model with a finely disaggregated age structure. Numbers of rotavirus gastroenteritis deaths and intussusception deaths in each week of age were calculated for all infants born in the year 2015 between birth and age 5·0 years, with and without restrictions on age at administration. Benefit–risk ratios (rotavirus gastroenteritis deaths prevented per excess intussusception death) and other indicators were calculated for two vaccination schedules currently recommended by WHO and 16 alternative schedules. Of these schedules, it was assumed that between one and three doses would be given; the first dose of the rotavirus vaccine would be co-administered with either BCG or diphtheria–tetanus–pertussis (DTP)1; and the second or third dose would be co-administered with either DTP1, DTP2, DTP3, or measles (Meas)1.

**Findings:**

A three-dose schedule co-administered with DTP (without age restrictions) could prevent about 74 000 (95% uncertainty interval 59 000–100 000) rotavirus gastroenteritis deaths (38% reduction) and could lead to 201 (77–550) excess intussusception deaths (1·4% increase) compared with no vaccination, resulting in a benefit–risk ratio of 369:1 (160:1–895:1). The benefit–risk ratio was most favourable when the relative risk of intussusception was assumed to decline with the national under-5 mortality rate (2386:1) and least favourable with pessimistic assumptions about access to hospital for intussusception treatment (168:1). Schedules that involve giving the first dose with BCG and the second with DTP1 had the fewest excess intussusception deaths and most favourable benefit–risk ratios.

**Interpretation:**

Rotavirus vaccines have a favourable benefit–risk profile in LMICs. Neonatal schedules have the potential to prevent more rotavirus gastroenteritis deaths and cause fewer excess intussusception deaths than the schedules currently recommended by WHO, but more efficacious rotavirus vaccines would be needed to achieve more substantial mortality reduction benefits.

**Funding:**

Bill & Melinda Gates Foundation.

## Introduction

Infant rotavirus vaccines have led to substantial reductions in hospital admissions for gastroenteritis,^1^ but some studies have reported an elevated risk of intussusception, a rare bowel disorder.^2,3^ In 2012, modelled estimates of the potential benefits and risks of introducing rotavirus vaccines into the national immunisation programmes of 158 countries provided reassurance about the highly positive benefit–risk profile of these vaccines.^4^ This analysis also informed a WHO recommendation to remove the manufacturers’ age restrictions for vaccine administration in settings where the mortality reduction benefits of late vaccination greatly exceeded the intussusception risk.^5^ Since the 2012 analysis, estimates of the number of rotavirus gastroenteritis deaths in children younger than 5 years (without vaccination) have decreased from about 450 000 in 2008 to about 200 000 in 2015.^6^ The evidence for several other modelling parameters has also been substantially strengthened, including new estimates of the efficacy of live oral rotavirus vaccines by duration of follow-up;^7^ the age distribution of rotavirus gastroenteritis hospital admissions in children younger than 5 years;^8^ intussusception incidence rates, age distributions, and case fatality ratios in children younger than 5 years;^9^ and the relative risk (RR) of intussusception 1–7 days and 8–21 days after administration of the first two doses of rotavirus vaccination,^10^ including the first risk estimates from high-mortality settings.^11,12^ Hence, it is now possible to produce updated and more robust benefit–risk estimates.

Research in context

**Evidence before this study**

We searched PubMed (“rotavirus vaccines” AND “risk assessment”) without language restrictions, from database inception until Sept 5, 2019, and identified 11 studies on benefit–risk assessments published between May, 2009, and September, 2018. In eight studies of low-mortality countries (Australia, England, two in France, Japan, the Netherlands, Singapore, and the USA), deaths from rotavirus or intussusception were very rare. Three studies evaluated multiple higher mortality countries. In the most recent study, published in 2012, the number of gastroenteritis deaths prevented per excess intussusception death was 371:1 for an age-restricted schedule co-administered with diphtheria–tetanus–pertussis, indicating a highly positive benefit–risk profile for rotavirus vaccination. This study also informed a WHO recommendation to remove the manufacturers’ age restrictions for vaccination given that the benefits of preventing additional rotavirus mortality from later vaccination greatly exceeded the intussusception risks (a benefit–risk ratio of 154:1).

**Added value of this study**

This analysis, based on updated evidence, provides continued reassurance about the highly positive benefit–risk profile of infant rotavirus vaccines. It provides modelled estimates of the number of rotavirus gastroenteritis and intussusception deaths for a range of different rotavirus vaccination schedules and scenarios. These results can be scrutinised at the national level and used to inform decision making in low-income and middle-income countries.

**Implications of all the available evidence**

This analysis supports the WHO recommendations to include rotavirus vaccination in national immunisation programmes and to remove age restrictions in countries where the mortality reduction benefits of late vaccination greatly exceed the risks. Schedules that involve giving the first dose with BCG and the second dose with diphtheria–tetanus–pertussis 1 could further increase effect and reduce risks, but more studies are required to assess their safety. There is a need for improved estimates of the proportion of children with timely access to intussusception treatment.

The scale of benefits and risks due to rotavirus vaccination will depend on the choice of vaccination schedule. For programmatic and economic reasons, rotavirus vaccines are currently co-administered with diphtheria–tetanus–pertussis (DTP)-containing vaccines in the first 6 months of life. More than half the countries in the world have introduced rotavirus vaccines. Countries either give two doses with DTP1 and DTP2, or three doses with DTP1, DTP2, and DTP3 as per current WHO recommendations. Randomised controlled trials using these schedules have shown high and durable rotavirus vaccine efficacy in high-income countries but lower and less durable efficacy in low-income and middle-income countries (LMICs).^7^ These trials have stimulated interest in the potential value of a neonatal dose given at the same time as BCG, a booster dose given with the first dose of measles vaccine (Meas1), or both. A neonatal dose has the potential to prevent disease that occurs very early in life and has been shown to be highly efficacious in Indonesia when administered as part of a three-dose schedule.^13^ A booster dose has the potential to mitigate the effects of waning rotavirus vaccine protection and has been shown to be non-interfering and immune-boosting in trials.^14,15^ The optimal number and timing of doses (concurrent with different combinations of BCG, DTP1, DTP2, DTP3, and Meas1) will depend on several factors, including the balance of benefits to risks.

The aim of this study was to investigate the potential mortality reduction benefits and intussusception risks of current rotavirus vaccination schedules in LMICs and to explore whether alternative schedules could have advantages.

## Methods

### Model design

All countries with a gross national income per capita of less than US$12 236 in the 2018 fiscal year were included in the model.^16^ An Excel-based static cohort model with a finely disaggregated age structure (weeks of age up to 5 years) was used to calculate potential benefits and risks of vaccination.^17^ In each country, the model was used to calculate numbers of doses administered, fully vaccinated infants, rotavirus gastroenteritis deaths, intussusception cases, and intussusception deaths expected to occur among all infants born in the year 2015 from birth to age 5.0 years. Estimated benefits (rotavirus gastroenteritis deaths averted) and risks (excess intussusception cases and deaths) were calculated by comparing each schedule scenario to a scenario with no rotavirus vaccination. The incremental benefits and risks of moving from age-restricted schedules to age-unrestricted schedules were also calculated. The primary outcome measure was the benefit–risk ratio of rotavirus vaccination (number of rotavirus gastroenteritis deaths prevented per excess intussusception death). Other indicators were the percent reduction in rotavirus gastroenteritis deaths, percent increase in intussusception deaths, number of fully vaccinated infants per excess intussusception case, and number of rotavirus gastroenteritis deaths prevented per dose administered.

### Vaccination schedule scenarios

Several licensed rotavirus vaccines are available nowadays, but evidence from comparisons in the same populations is insufficient to show conclusive superiority of one brand over another in terms of vaccine efficacy, effectiveness, or impact,^3,18–20^ or intussusception risks.^3,21^ Thus, all currently licensed vaccines were assumed to be equivalent in these respects. To restrict the vaccine schedules considered to a manageable number, it was assumed that between one and three doses would be given; the first dose of the rotavirus vaccine would be co-administered with either BCG or DTP1; and the second or third dose would be co-administered with either DTP1, DTP2, DTP3, or Meas1. This approach resulted in 18 possible schedules (table 1). For the first 11 schedule options (all primary dose schedules), scenarios with and without strict adherence to age restrictions were modelled (first dose administered before 15 weeks of age; last dose delivered before 32 weeks of age).

**Table 1:** List of schedules evaluated for co-administration of rotavirus and other vaccines

	Neonatal dose schedule	Booster dose schedule	Age-restricted scenario^*^	Age-unrestricted scenario
BCG	Yes	No	Yes	Yes
DTP1	No	No	Yes	Yes
BCG plus DTP1	Yes	No	Yes	Yes
BCG plus DTP2	Yes	No	Yes	Yes
BCG plus DTP3	Yes	No	Yes	Yes
DTP1 plus DTP2†	No	No	Yes	Yes
DTP1 plus DTP3	No	No	Yes	Yes
BCG plus DTP1 plus DTP2‡	Yes	No	Yes	Yes
BCG plus DTP1 plus DTP3	Yes	No	Yes	Yes
BCG plus DTP2 plus DTP3	Yes	No	Yes	Yes
DTP1 plus DTP2 plus DTP3†	No	No	Yes	Yes
BCG plus Meas1	Yes	Yes	No	Yes
DTP1 plus Meas1	No	Yes	No	Yes
BCG plus DTP1 plus Meas1	Yes	Yes	No	Yes
BCG plus DTP2 plus Meas1	Yes	Yes	No	Yes
BCG plus DTP3 plus Meas1	Yes	Yes	No	Yes
DTP1 plus DTP2 plus Meas1§	No	Yes	No	Yes
DTP1 plus DTP3 plus Meas1	No	Yes	No	Yes

DTP=diphtheria–tetanus–pertussis. Meas=measles vaccine.

* First vaccination before 15 weeks of age; final vaccination before 32 weeks of age.

† Schedules recommended by WHO; the three-dose schedule has been evaluated in efficacy trials for Rotarix, RotaTeq, ROTAVAC, ROTASIIL, and RV3-BB; the two-dose schedule has been evaluated in Rotarix efficacy studies.7

‡ Schedule evaluated in RV3-BB efficacy study in Indonesia.^13^

§ Schedule evaluated in Rotarix immunogenicity studies in Bangladesh^15^ and Mali.^14^

### Potential benefits of rotavirus vaccination

For a given country and week (w) of age, the number of rotavirus gastroenteritis deaths was calculated as:

$$PY\times M\times A_{w}\times \left(1-V_{w}\right)$$

where PY × M × A_w_ is the number of rotavirus gastroenteritis deaths in week w of age; V_w_ is the direct effect of vaccination in week w of age; PY is the number of person-years lived between birth and age 5·0 years in the 2015 birth cohort;^22^ M is the rotavirus gastroenteritis mortality rate per 100 000 population per year among children younger than 5 years before the introduction of rotavirus vaccination; A_w_ is the proportion of rotavirus deaths in children younger than 5 years in week w of age.

The direct effect of vaccination in each week of age (V_w_) was calculated as:

$$C_{3w}\times E_{3w}+\left(C_{2w}-C_{3w}\right)\times E_{2w}+(C_{1w}-C_{2w})\times E_{1w}$$

where C_3w_ × E_3w_ is the direct effect contributed by infants that received all three doses; (C_2w_ – C_3w_) × E_2w_ the direct effect contributed by infants that received only two doses; (C_1w_ – C_2w_) × E_1w_ the direct effect contributed by infants that received only one dose; C_1w_, C_2w_, and C_3w_ are coverage estimates for the first three doses of rotavirus vaccination in week w of age, each adjusted for age restrictions if applicable; and E_1w_, E_2w_, and E_3w_ are efficacy estimates for the first three doses of rotavirus vaccination in week w of age, each adjusted for waning since the time of vaccination. All parameters used to estimate benefits are defined in the appendix (pp 1, 2, 16).

### Potential risks of rotavirus vaccination

For a given country, dose d, and week w of age, the number of excess (vaccine-related) intussusception events (eg, cases or deaths) was calculated as:

$$P\times \left(C_{d,w}-C_{d,w-1}\right)\times [B_{w}\times \left(RR_{d,1-7}-1\right)+B_{w+1}\times (RR_{d,8-14}-1)+B_{w+2}\times \left(RR_{d,15-21}-1\right)]$$

where P × (C_d, w_ – C_d, w – 1_) is the number of infants receiving dose d in week w of age, B_w_ × (RR_d, 1 – 7_ – 1) is the rate of intussusception 1–7 days after dose d in week w of age, B_w + 1_ × (RR_d, 8 – 14_ – 1) is the rate of intussusception 8–14 days after dose d in week w of age, B_w + 2_ × (RR_d, 15 – 21_ – 1) is the rate of intussusception 15–21 days after dose d in week w of age, and P is the mid-year population for the relevant single year of age; C_d, w_ and C_d, w – 1_ are cumulative coverage estimates for dose d in weeks w and w–1, respectively; B_w_, B_w + 1_, and B_w + 2_ are the background rates of intussusception events in weeks w, w + 1, and w + 2 of age, respectively; and RR_d, 1 – 7_, RR_d, 8 – 14_, RR_d, 15 – 21_ are the relative risks of vaccine-related intussusception associated with dose d in the periods 1–7 days, 8–14 days, and 15–21 days after vaccination, respectively. RRs were assumed to be only dose-dependent, with no independent effect of age of vaccine administration. All parameters used to estimate risks are defined in the appendix (pp 3, 4, 16).

### Uncertainty and scenario analysis

Deterministic central estimates (ie, best estimates for each input parameter) and probabilistic 95% uncertainty intervals (UIs) were calculated for 11 age-restricted schedules and 18 age-unrestricted schedules. All input parameters and their distributions are shown in the appendix (p 16). Central estimates were also calculated for six what-if scenarios: RRs of intussusception varying with under-5 mortality (figure, appendix p 12); double the RR of intussusception for the first dose when given after 15 weeks of age; vaccine efficacy and waning equivalent to low-mortality settings; less rapid waning efficacy (based on a power function described in detail elsewhere);^7^ less rapid waning efficacy for all primary doses administered as part of a neonatal schedule (appendix p 14); and pessimistic access to hospital for intussusception cases (based on the proportion of children with 2 h access to a public hospital).^28^

**Figure:**
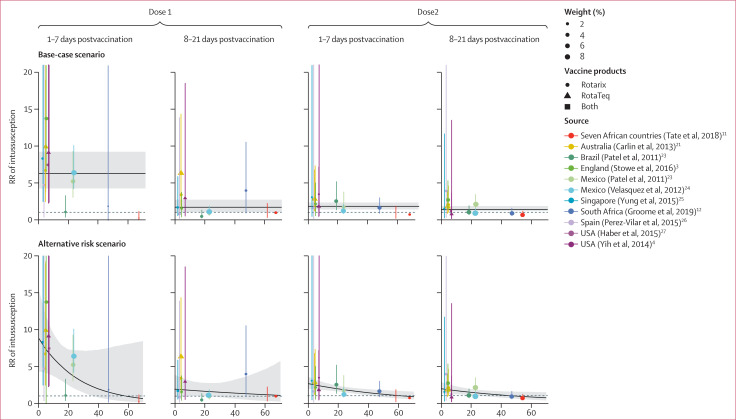
RR of intussusception 1–7 days and 8–21 days after one and two doses of rotavirus vaccine in base-case risk scenario and alternative risk scenario The four panels on the top row represent the base-case risk scenario (relative risks [RRs] do not vary with under-5 mortality rate). The four panels on the bottom row represent an alternative risk scenario and assume RRs vary with the national under-5 mortality rate. Solid black lines indicate the median estimate and shaded grey areas represent 95% CIs. Circles and vertical lines represent the RR and 95% CIs reported for each individual self-controlled case-series data point. The size of the circle reflects the inverse of the variance (weight attributed to each study). See appendix (p 12) for more details about the fitting methods used. RR=relative risk.

Results for each schedule option reflect the totals expected in all 135 LMICs if all countries used the same schedule. However, to illustrate the maximum potential direct effect of the current live oral vaccines, separate totals for the model outcomes were calculated to show results if each country used the schedule predicted to have the highest reduction in rotavirus gastroenteritis deaths.

### Statistical analysis

We did a random effects meta-analysis of data from self-controlled case-series (SCCS) studies to calculate pooled RRs of intussusception by dose and period. The inverse of the variance was used to weight each study. Very few studies reported RRs for the periods 8–14 days and 15–21 days, so we calculated RRs for the period 8–21 days, and applied these RRs to both periods (ie, RR_d, 8 – 14_ and RR_d, 15 – 21_; table 2, appendix p 5). In one scenario, we assumed the RRs would vary with the national under-5 mortality rate, and this was done by fitting a generalised linear random effects model with a log-link function to the SCCS data using Markov Chain Monte Carlo with Gibbs sampling, and assuming a linear relationship between the log mean RR and under-5 mortality rate. The inverse of the variance was again used to weight each study. We compared the goodness of fit (Deviance Information Criterion values) for RRs that did and did not vary with under-5 mortality (figure, appendix p 12). We also ran a random effects meta-analysis using data from case control studies to calculate the relative effectiveness of 1 dose compared with 2 or 3 doses of rotavirus vaccination (appendix p 15). Random effects were used in all meta-analyses due to heterogeneity between the different study populations.

**Table 2:** Pooled random effects meta-analysis of the relative risk of intussusception in the periods 1–7 days and 8–21 days after doses 1 and 2 of rotavirus vaccination

	w	Vaccine	After dose 1						After dose 2					
			1–7 days			8–21 days			1–7 days			8–21 days		
			Relative risk	Lower 95% uncertainty interval limit	Upper 95% uncertainty interval limit	Relative risk	Lower 95% uncertainty interval limit	Upper 95% uncertainty interval limit	Relative risk	Lower 95% uncertainty interval limit	Upper 95% uncertainty interval limit	Relative risk	Lower 95% uncertainty interval limit	Upper 95% uncertainty interval limit
**Relative risk per study**														
Yung et al^25^ (Singapore)	2·6	Rotarix	8·36	2·42	28·96	1·72^*^	0·51	5·88	3·09	0·41	23·37	1·54	0·20	11·69
Perez-Vilar et al^26^ (Spain)	3·5	Rotarix or RotaTeq	4·70	0·30	74·10	0·80	0·10	13·90	1·60	0·10	32·30	3·90	0·30	44·00
Carlin et al^21^ (Australia)	4·6	Rotarix	6·76	2·40	19·01	3·45	1·33	8·94	2·84	1·10	7·34	2·11	0·97	4·62
Carlin et al^21^ (Australia)	4·6	RotaTeq	9·89	3·70	26·42	6·32	2·78	14·37	2·81	1·16	6·80	1·77	0·81	3·88
Stowe et al^2^ (England)	4·7	Rotarix	13·81†	6·44	28·32	1·59	0·34	3·75	2·20	0·50	5·02	2·77	1·36	5·32
Haber et al^27^ (USA)	7·0	Rotarix	7·50	2·30	24·60	··	··	··	2·40‡	0·80	7·50	··	··	··
Yih et al^3^ (USA)	7·0	Rotarix	··	··	··	··	··	··	3·50	0·50	25·10	1·21§	0·11	13·53
Yih et al^3^ (USA)	7·0	RotaTeq	9·10	2·20	38·60	2·93§	0·46	18·55	1·80	0·40	7·20	0·76§	0·16	3·67
Haber et al^29^ (USA)	7·0	RotaTeq	3·75‡	1·90	7·39	··	··	··	1·43‡	0·85	2·40	··	··	··
Escolano et al^30^ (worldwide)	>7·0	RotaTeq	3·45‡	1·84	6·55	0·91‡	0·51	1·62	1·63‡	0·86	3·13	1·07‡	0·63	1·80
Patel et al^23^ (Brazil)	18·4	Rotarix	1·10	0·30	3·30	0·51¶	0·20	1·33	2·60	1·30	5·20	1·12	0·65	1·93
Patel et al^23^ (Mexico)	23·2	Rotarix	5·30	3·00	9·30	0·99¶	0·52	1·91	1·80	0·90	3·80	2·20	1·40	3·45
Velazquez et al^24^ (Mexico)	23·2	Rotarix	6·49	4·17	10·09	1·19||	0·78	1·83	1·29	0·80	2·11	1·00||	0·66	1·52
Groome et al^12^ (South Africa)	47·0	Rotarix	1·86^**^	0·09	37·09	4·01	0·87	10·56	1·71	0·83	3·01	0·96	0·52	1·60
Tate et al^11^ (Africa)	67·0	Rotarix	0·25	0·00	1·16	1·01	0·26	2·24	0·76	0·16	1·87	0·74	0·39	1·20
**Base-case risk scenario**														
Pooled RR	··	··	6·26	4·25	9·22	1·69	1·05	2·72	1·82	1·41	2·34	1·37	1·03	1·84
*l*^2^	··	··	39·0%	··	··	61·7%	··	··	0·0%	··	··	44·9%	··	··
p value	··	··	0·081	··	··	0·0036	··	··	0·76	··	··	0·046	··	··
**Scenario with overlapping data points**														
Pooled RR	··	··	5·52	3·93	7·75	1·56	1·02	2·40	1·75	1·41	2·16	1·33	1·03	1·73
*l*^2^	··	··	43·0%	··	··	61·5%	··	··	0%	··	··	41·7%	··	··
p value	··	··	0·044	··	··	0·0026	··	··	0·86	··	··	0·057	··	··

RR=relative risk.

* No intussusception cases occurred in the period 8–21 days, so the risk was calculated as: exp ([ln (RR1 – 21) – (ln (RR1 – 7) × (7/22))]/(15/22)).

† RR based on method that included historical cases to adjust for age.

‡ Indicates data points that were excluded from the main analysis because a data point was available from a more systematic study for the same population, time period, brand, dose, or risk period; two studies from Mexico reflected different populations so were both included in the main analysis; pooled RR estimates that allowed for overlapping data points indicated slightly lower risk than the pooled RRs for the base-case scenario.

§ RR in the period 8–21 days was calculated as: exp ([ln (RR1–21) – (ln (RR1–7) × (7/22))]/(15/22)).

|| RR in the period 7–30 days was used as a proxy for 8–21 days and was calculated as: exp ([ln (RR0 – 30) – (ln (RR0 – 6) × (7/31))]/(24/31)).

¶ RR in the period 8–21 days was calculated as: exp (average [ln (RR8 – 14), ln (RR15–21)]).

** No intussusception cases occurred in the 1–7-day period after dose 1 so the risk in this period was calculated as: exp ([ln (RR1 – 21) – ((15/22) × ln (RR8 – 21))]/(7/22)).

### Role of the funding source

The funder of the study had no role in study design, data collection, data analysis, data interpretation, or writing of this report. The corresponding author had full access to all the data in the study and had final responsibility for the decision to submit for publication.

## Results

Mortality reduction benefits and intussusception risks were calculated for 31 low-income, 51 lower-middle-income, and 53 upper-middle-income countries. In all 135 LMICs (a combined birth cohort of about 60 million children), around 194 000 rotavirus gastroenteritis deaths (95% UI 159 000–257 000; tables 3, 4) were estimated in children younger than 5 years without vaccination. The model predicted that 18–41% of these deaths could be prevented by rotavirus vaccination, depending on the schedule used. Around 14 500 background intussusception deaths (8000–27 000; tables 3, 4) were estimated in children younger than 5 years. In the base-case vaccination scenario (ie, with the best assumptions), the model predicted that no more than 213 additional deaths (about 1·5% increase; table 3) would be caused by rotavirus vaccination for any schedule evaluated.

**Table 3:** Potential benefits and risks of alternative rotavirus vaccination schedule options in 135 low-income and middle-income countries

	Doses		Rotavirus gastroenteritis deaths			Intussusception deaths			Summary indicators				
	Total doses (millions)^*^	Fully vaccinated infants (millions)	n	Number averted (*vs* no vaccine)	Number averted (*vs* age-restricted)	n	Excess number (*vs* no vaccine)	Excess number (*vs* age-restricted)	Reduction in rotavirus gastroenteritis deaths (%)	Doses per rotavirus gastroenteritis death averted	Number of fully vaccinated infants per excess intussusception case	Rotavirus gastroenteritis deaths averted per excess intussusception death (95% uncertainty interval)	Incremental rotavirus gastroenteritis deaths averted per excess intussusception death *vs* agerestricted (95% uncertainty interval)
No vaccine	0	0	194 471	··	··	14 478	··	··	··	··	··	··	··
Age-restricted (primary dose schedules)													
BCG	109	103	158 527	35 945	··	14 505	27	··	18·5%	3032	643 556	1325 (540–3266)	··
DTP1	90	86	160 292	34 179	··	14 549	71	··	17·6%	2646	133 349	481 (196–1163)	··
BCG plus DTP1	217	103	133 584	60 887	··	14 530	53	··	31·3%	3564	220 468	1157 (474–3306)	··
BCG plus DTP2	214	100	131 626	62 845	··	14 562	85	··	32·3%	3404	117 145	743 (312–2186)	··
BCG plus DTP3	196	83	134 629	59 842	··	14 569	91	··	30·8%	3276	91 528	655 (288–1774)	··
DTP1 plus DTP2†	180	86	136 941	57 531	··	14 600	122	··	29·6%	3142	75 069	471 (201–1242)	··
DTP1 plus DTP3	170	76	138 001	56 470	··	14 612	134	··	29·0%	3020	59 745	421 (184–1089)	··
BCG plus DTP1 plus DTP2	322	100	127 733	66 739	··	14 530	53	··	34·3%	4822	214 021	1269 (542–3316)	··
BCG plus DTP1 plus DTP3	304	83	125 308	69 164	··	14 530	53	··	35·6%	4394	177 465	1315 (560–3436)	··
BCG plus DTP2 plus DTP3	300	83	126 718	67 753	··	14 562	85	··	34·8%	4441	97 152	801 (333–2336)	··
DTP1 plus DTP2 plus DTP3†	260	76	131 986	62 485	··	14 600	122	··	32·1%	4173	66 530	512 (218–1338)	··
Age-unrestricted (primary dose schedules)													
BCG	114	108	156 279	38 192	2247	14 524	46	19	19·6%	2993	373 252	828 (346–1977)	118 (56–246)
DTP1	117	111	153 245	41 227	7048	14 618	140	69	21·2%	2842	71 459	294 (125–684)	102 (48–217)
BCG plus DTP1	227	107	130 147	64 324	3438	14 546	68	16	33·1%	3532	186 572	940 (394–2474)	218 (108–476)
BCG plus DTP2	225	105	128 034	66 438	3592	14 583	106	21	34·2%	3393	104 475	630 (265–1733)	172 (84–363)
BCG plus DTP3	223	103	128 592	65 879	6037	14 596	119	27	33·9%	3387	89 036	555 (241–1443)	221 (105–469)
DTP1 plus DTP2†	230	107	126 649	67 823	10 292	14 679	201	79	34·9%	3400	47 707	338 (145–831)	131 (61–280)
DTP1 plus DTP3	226	104	127 011	67 460	10 990	14 691	213	79	34·7%	3365	43 032	316 (137–768)	139 (65–292)
BCG plus DTP1 plus DTP2	338	105	123 843	70 628	3890	14 545	67	15	36·3%	4788	187 788	1049 (455–2544)	264 (122–576)
BCG plus DTP1 plus DTP3	335	103	120 044	74 428	5264	14 546	68	16	38·3%	4512	180 330	1092 (453–2722)	338 (158–724)
BCG plus DTP2 plus DTP3	334	103	121 869	72 603	4850	14 583	105	21	37·3%	4601	102 349	689 (291–1881)	234 (115–490)
DTP1 plus DTP2 plus DTP3†	339	104	120 323	74 148	11 663	14 678	201	79	38·1%	4590	46 222	369 (160–895)	148 (69–317)
Age-unrestricted (booster dose schedules)													
BCG plus Meas1	222	102	135 675	58 796	··	14 563	85	··	30·2%	3783	122 994	689 (293–1592)	··
DTP1 plus Meas1	225	103	133 285	61 187	··	14 656	179	··	31·5%	3691	49 466	343 (146–797)	··
BCG plus DTP1 plus Meas1	335	102	117 652	76 819	··	14 546	68	··	39·5%	4360	178 757	1125 (466–2937)	··
BCG plus DTP2 plus Meas1	332	102	116 970	77 501	··	14 583	105	··	39·9%	4294	101 289	735 (306–2024)	··
BCG plus DTP3 plus Meas1	329	101	119 377	75 094	··	14 596	118	··	38·6%	4389	87 531	634 (271–1649)	··
DTP1 plus DTP2 plus Meas1	338	103	115 528	78 944	··	14 678	201	··	40·6%	4292	45 610	393 (168–974)	··
DTP1 plus DTP3 plus Meas1	333	102	117 757	76 714	··	14 691	213	··	39·4%	4358	42 111	360 (155–871)	··

DTP=diphtheria–tetanus–pertussis. Meas=measles vaccine. Numbers are the totals expected in all 135 low-income and middle-income countries if all countries used the same schedule.

* Assumes 5% wastage for all doses.

† Current schedule options recommended by WHO; other options should be interpreted with caution until more evidence is established on their safety and efficacy.

**Table 4:** Benefits and risks of removing age restrictions for rotavirus vaccine administration in 135 low-income and middle-income countries for a three-dose rotavirus schedule co-administered with diphtheria–tetanus–pertussis vaccine

	Rotavirus gastroenteritis deaths^*^			Intussusception deaths^*^			Summary indicators^*^	
	n	Number averted *vs* no vaccine (95% uncertainty interval)	Reduction in deaths *vs* no vaccine (%)	n	Excess number *vs* no vaccine	Increase *vs* no vaccine (%)	Number of fully vaccinated infants per excess intussusception case	Rotavirus gastroenteritis deaths averted per excess intussusception death
No vaccine	194 471 (158 603–257 080)	··	··	14 478 (8028–27 463)	··	··	··	··
Age-restricted scenario	131 986 (106 800–176 694)	62 485 (47 895–83 238)	32·1% (26·1–37·1)	14 600 (8112–27 709)	122 (44–322)	0·8% (0·3–1·7)	66 530 (22 330–187 422)	512 (218–1338)
Age-unrestricted scenario	120 323 (97 540–159 450)	74148 (59 362–100 227)	38·1% (33·1–42·9)	14 678 (8165–27 807)	201 (77–550)	1·4% (0·6–2·8)	46 222 (14 647–123 585)	369 (160–895)
Age-unrestricted *vs* age-restricted	··	11 663 (6522–22 532)	6·0% (3·4–10·5)	··	79 (29–236)	0·5% (0·2–1·2)	25 369 (8579–63 401)	148 (69–317)

Data estimated for children younger than 5 years.

* 95% uncertainty intervals represent the 2.5th and 97.5th percentiles of probabilistic simulations; see appendix (p 16) for more details.

For age-unrestricted schedules co-administered with DTP, the predicted reduction in rotavirus gastroenteritis deaths was 20% for one-dose schedule, 34% for two-dose schedule, and 38% for three-dose schedule (table 3). The dose efficiency (number of doses required to prevent each death) was circa 2800, 3400, and 4600, respectively (table 3). A three-dose schedule could prevent about 74 000 (59 000–100 000; table 4) rotavirus gastroenteritis deaths (38% reduction) and lead to 201 (77–550) excess intussusception deaths (1·4% increase; table 4) compared with no vaccination, resulting in a benefit–risk ratio of 369:1 (160:1–895:1). Infants who received their first dose before 15 weeks of age and their last dose before 32 weeks of age had a benefit–risk ratio of 512:1 (218:1–1338:1) compared with 148:1 (69:1–317:1) among infants vaccinated after these ages (table 4). Compared with an age-restricted schedule, a schedule without age restrictions was associated with about 12 000 (7000–23 000) fewer rotavirus gastroenteritis deaths and 79 (29–236) more intussusception deaths (table 4). Among children vaccinated outside the recommended age range, the benefit–risk ratio exceeded 100:1 in 102 (76%) LMICs, but in 14 countries (Algeria, Argentina, Equatorial Guinea, Iraq, Libya, Mauritius, Moldova, Saint Lucia, Saint Vincent and the Grenadines, Samoa, Syria, Tonga, Vanuatu, and Vietnam) the ratio was below 50:1 (appendix p 17). The model estimated one excess case of intussusception per 46 000 fully vaccinated infants (15 000–124 000). The risk was lower for children vaccinated inside the age windows (one case per 67 000 [22 000–187 000]) than those vaccinated outside (one case per 25 000 [9000–63 000]; appendix p 17).

Neonatal schedules that involve giving the first two doses as early as possible (ie, with BCG and DTP1) had the fewest excess intussusception deaths and favourable benefit–risk ratios compared with other schedules (table 3). A third dose given with Meas1 (ie, BCG plus DTP1 plus Meas1) was predicted to prevent more rotavirus gastroenteritis deaths without negatively impacting the benefit–risk ratio.

If each country were to use the age-unrestricted schedule with the highest predicted reduction in rotavirus gastroenteritis deaths, there would be more rotavirus gastroenteritis deaths averted (about 81 000 *vs* 74 000), fewer intussusception deaths (148 *vs* 201), and a more favourable benefit–risk ratio (550:1 *vs* 369:1) compared with a standard age-unrestricted schedule given with DTP (appendix p 21).

Schedules incorporating a neonatal dose did not have the highest predicted reduction in rotavirus gastroenteritis deaths if coverage of BCG was zero (eg, Grenada, Lebanon, Suriname); coverage of BCG was substantially lower than DTP1 (eg, Ethiopia, Indonesia, South Africa); or the overall combination of input parameters (rotavirus gastroenteritis age distribution, coverage, timeliness, efficacy, and waning) led to marginally more rotavirus gastroenteritis deaths prevented by infant schedules (eg, Bangladesh). A booster dose schedule did not have the highest predicted reduction in rotavirus gastroenteritis deaths if age restrictions were applied, as very few booster doses are administered before 32 weeks of age; most rotavirus gastroenteritis deaths were in very young ages (eg, Afghanistan, Angola, Pakistan); or the efficacy of primary doses was assumed to be high and durable (eg, Brazil, Vietnam).

Assuming a gradient of risk consistent with under-5 mortality (figure) had a far more favourable benefit–risk ratio than the base-case risk assumptions (2386:1 *vs* 369:1 for a three-dose age-unrestricted schedule co-administered with DTP). In this scenario, there were zero excess intussusception cases in 29 (21%) of 135 LMICs and fewer than 35 excess intussusception deaths each year for any schedule evaluated (appendix p 25). If the RR of intussusception for the first dose was doubled after 15 weeks of age rather than constant with age (appendix p 30), the benefit–risk for children vaccinated outside the recommended age range would be much lower (66:1 *vs* 148:1). Assuming vaccine efficacy and waning equivalent to low-mortality settings led to a much higher reduction in rotavirus gastroenteritis deaths (75% *vs* 42%) if all countries were assumed to adopt the schedule with the highest reduction in rotavirus gastroenteritis deaths (appendix p 36). A scenario with more durable efficacy improved the benefit–risk ratio (383:1 *vs* 369:1 for a three-dose age-unrestricted schedule co-administered with DTP) as expected (appendix p 40). If doses given as part of a neonatal schedule were assumed to have double the mean duration of protection, then neonatal schedules had the highest predicted effect in most countries (appendix p 46). In a scenario with very pessimistic assumptions about access to intussusception treatment there were less favourable benefit–risk ratios (168:1 *vs* 369:1 for a three-dose age-unrestricted schedule co-administered with DTP) but no more than 480 excess intussusception deaths each year for any schedule evaluated (appendix p 50).

## Discussion

For the current live oral vaccines and schedules recommended by WHO, our model estimated one excess case of intussusception per approximately 46 000 fully vaccinated individuals. This risk is more favourable than the risk (one excess case per <10 000 fully vaccinated individuals) associated with RotaShield (Wyeth-Ayerst, Philadelphia, PA, USA), an early rotavirus vaccine that was withdrawn from the market in the USA,^31^ but less favourable than the risk associated with other vaccines, such as BCG vaccine (one excess case of disseminated BCG disease per >200 000 fully vaccinated individuals)^32^ and oral polio vaccine (one excess case of vaccine associated paralytic poliomyelitis per >700 000 fully vaccinated individuals).^33^ However, any estimates of the potential risk should also be considered in the context of potential benefits, and this analysis continues to provide reassurance about the positive benefit–risk profile of the currently licensed rotavirus vaccines. The central estimate of the benefit– risk ratio for an age-unrestricted schedule co-administered with DTP (369:1) is very similar to the previous estimate (371:1),^4^ with the benefits of rotavirus vaccine introduction (74 000 rotavirus gastroenteritis deaths averted) still greatly exceeding the risk (201 intussusception deaths caused).

In the previous analysis removing age restrictions from a standard infant schedule of rotavirus co-administered with DTP was estimated to reduce gastroenteritis deaths by about 47 000 per year and increase intussusception deaths by about 300 per year.^4^ In the new analysis, the equivalent estimates are much lower (about 12 000 rotavirus gastroenteritis deaths prevented and 79 excess intussusception deaths) but the incremental benefit–risk ratio is very similar (148:1 *vs* 154:1). The new analysis, therefore, still supports the WHO recommendation to remove age restrictions in countries where the benefit would greatly exceed the risk.^5^ The predicted mortality reduction benefits were much lower because prevaccination estimates of rotavirus mortality are now substantially lower.^6^ The number of excess intussusception deaths was also much lower because the median age of intussusception from updated estimates was higher,^9^ resulting in fewer background intussusception cases around the time the first rotavirus dose was administered. In both analyses, it was assumed that introducing or removing age restrictions for rotavirus vaccines would not lead to better or worse adherence to schedules, but more evidence is needed from postintroduction studies to confirm this assumption. In a small number of countries (eg, Argentina, Vietnam), the benefit–risk ratio of introduction or the removal of age restrictions was less obviously favourable. In these countries, the input assumptions should be carefully reviewed by local experts, and a fuller assessment of the broader economic benefits of vaccination should be considered. The UNIVAC vaccine decision support model used in this analysis has been designed for use at country level, and is available to download by Ministries of Health and other national stakeholders who wish to better understand the national estimates and explore scenarios.^17^

Only RR estimates based on the SCCS method were considered because they are based on large numbers of postlicensure vaccine recipients. It is important to note that only two SCCS studies, both evaluating Rotarix (GlaxoSmithKline Biologicals, London, UK) in Africa,^11,12^ have evaluated the postlicensure risk of intussusception in high-mortality settings, and in both studies the RR was low and the association was not statistically significant for any dose or period. In a scenario based on a gradient of risk consistent with national under-5 mortality, the model predicted far more favourable benefit–risk ratios than the base-case analysis. However, more evidence is needed from other parts of the world before a risk can be completely excluded in high-mortality countries. Another important consideration is whether rotavirus vaccination might simply be triggering intussusception events that would otherwise occur in the same children at a later date.^34^

The RRs used in the model did not vary with age. However, since the RR is applied to the background age-specific incidence of intussusception, the absolute risk is still highly age-dependent. Should the RR increase at older ages of vaccination,^35^ then removing age restrictions would be less favourable in most countries, unless the first dose is co-administered with BCG rather than DTP1. Assuming a risk relative to (rather than independent of) the background incidence of intussusception will always favour neonatal schedules, but this advantage is yet to be shown in a large number of postlicensure vaccine recipients. However, no excess risk was associated with RotaShield doses administered before 8 weeks and this effect is consistent with no excess risk associated with earlier administration of Rotarix (GlaxoSmithKline Biologicals, London, UK) in Africa (median age 6 weeks).^11^

For all 135 LMICs combined, the model estimated approximately 14 500 intussusception deaths for children younger than 5 years of age, but this number was very sensitive to the choice of proxy for access to treatment. DTP1 coverage was used because it provides a crude indicator of access to basic health services and is consistently reported for all LMICs. An alternative proxy based on the proportion of children with timely (2 h) access to public hospitals gave less favourable benefit– risk ratios. However, this scenario was probably too pessimistic because many intussusception cases in Africa are known to present to hospital more than 2 days after the onset of symptoms.^36^ In medium-mortality countries, access to care adjustments led to large increases in the background intussusception mortality relative to the (often very low) prevaccination rotavirus gastroenteritis mortality. This increase partly explains the modest benefit–risk ratio in some countries. For example, in Argentina, the model assumed that 5% of intussusception cases would not reach hospital (based on a proxy of 95% DTP1 coverage)^37^ and that 90% of these children would die. In these settings, an assumption of 100% access to treatment (consistent with countries in the low-mortality and very low-mortality strata) might be more appropriate. Improved estimates of treatment use for intussusception are needed.

Children at greater risk of rotavirus gastroenteritis mortality might be less likely to have access to routine vaccination programmes, and not accounting for this issue could have led to inflated numbers of rotavirus gastroenteritis deaths prevented.^38^ However, children with better access to vaccination might also have better access to intussusception treatment (and hence lower case fatality ratios for intussusception than the cohort as a whole), and not accounting for this scenario could also lead to inflated estimates of intussusception deaths in vaccinated children. Thus, adjustments for heterogeneities in risk and access are likely to balance out, to some extent at least.

This analysis highlights the potential value of a neonatal dose of rotavirus vaccination, in terms of both safety and impact. The safety of a neonatal dose is yet to be shown in a large-scale postlicensure setting, but this analysis predicts that fewer intussusception events would be caused by the vaccine if neonatal schedules were used. The only vaccine to have shown clinical efficacy using a neonatal schedule is the RV3-BB (Murdoch Children’s Research Institute, Melbourne, VA, Australia),^13^ and it is unclear whether Rotarix (GlaxoSmithKline Biologicals, London, UK), RotaSIIL (Serum Institute of India, Pune, India), RotaTeq (Merck, Kenilworth, NJ, USA), and ROTAVAC (Bharat Biotech, Hyderabad, India) would have similar results. It should be noted that when RotaShield was studied using a neonatal schedule, it was found to be safe and efficacious.^39^ In some countries, neonatal schedules did not have the highest predicted effect because coverage of BCG was substantially lower than DTP1. However, in these countries, the calculations of vaccine effect did not allow for opportunities to catch up on missed doses at later visits. Also, in some settings BCG might not be the best proxy for the coverage and timeliness of the neonatal dose of rotavirus vaccination. For example, in Indonesia, a neonatal schedule did not have the highest predicted effect because the target age for BCG is later than in other settings (can be given at any time in the first 2 months of life), and coverage of BCG is much lower than DTP1 (80% *vs* 95%).^37^ Thus, the neonatal dose of oral polio vaccine or hepatitis B vaccine would have been a better choice of proxy in this setting.

This analysis also highlights the potential benefit of a booster dose in mitigating the waning protection of rotavirus vaccines.^40^ A third dose of rotavirus vaccination co-administered with Meas1 was assumed to have the same efficacy (and waning) as a second dose co-administered with DTP. This assumption is consistent with a Rotarix immunogenicity study in Bangladesh, where seropositivity (immunoglobulin A titres ≥20 units per mL) increased from 53% to 70% when a third dose of Rotarix was administered concurrently with measles vaccine.^15^ A study in Mali also found an increase in immunoglobulin A titres and no negative effect on the immune response of other vaccines administered at the same visit (eg, measles, yellow fever).^14^ However, more evidence is needed on the safety, efficacy, and incremental cost-effectiveness of a booster dose, and until this evidence emerges, the results of this analysis should be interpreted with caution. Most of the excess cases associated with RotaShield were vaccinated as part of a catch-up campaign among older infants,^41^ although these cases were associated with the first and second dose, and no excess risk has been reported with the third dose of RotaTeq administered at about 6 months.^15^

If all 135 LMICs were to adopt the age-unrestricted schedule with the highest predicted reduction in rotavirus gastroenteritis deaths (including schedules with neonatal doses, booster doses, or both), 81 000 associated deaths could be averted (42% reduction) compared with 74 000 (38% reduction) for schedules co-administered with DTP. The risk would also be lower because these schedules typically involve administering the first dose with BCG when the background risk of intussusception is lower. However, if a rotavirus vaccine could be developed for LMICs with the same efficacy and duration of protection observed in high-income countries, a far greater number of deaths (about 146 000) could be averted (75% reduction). However, the predicted reduction in rotavirus gastroenteritis deaths is just one of many possible decision criteria that should be considered when selecting a schedule. Other criteria include the expected risks, benefit–risk, operational feasibility, cost, cost-effectiveness, and public acceptance. In this analysis, schedules that involve giving the first dose with BCG and the second dose with DTP1 had lower risk and favourable benefit–risk results, so warrant serious consideration. The choice of schedule should be informed by a detailed country-led review of inputs and careful consideration of the different trade-offs involved.

A transparent static cohort model was used to estimate the potential direct effects of vaccination by week of age. Inclusion of herd effects could make the benefit–risk ratios more favourable in some settings, but it would be challenging to obtain robust estimates of the scale and duration of these effects in each of the 135 LMICs. Transmission dynamic models calibrated to data from Niger^42^ and India^43^ have predicted a minimal contribution of indirect effects to overall vaccine effect, and although short-term herd effects have been observed in El Salvador,^44^ Ghana,^1^ Moldova,^1^ and Rwanda,^45^ no substantial herd effects were observed in Malawi,^46^ South Africa,^47^ Tanzania,^1^ and Zambia.^48^ In principle, transmission dynamic models could be used to anticipate the longer-term effect and relative advantages of different vaccination schedule options but these models will require access to good quality data on disease surveillance and social contact patterns in narrow age groups.

When RotaShield was removed from the market in the USA, ethicists argued that “the future of a potentially lifesaving vaccine for developing countries has been imperilled by its recent withdrawal”.^49^ A central argument was that inaction was not a morally neutral state, and that “if one is culpable for vaccine related deaths, then one is also culpable for deaths caused by withholding the vaccine”.^49^ However, it is also important to consider how individual families and caregivers perceive potential benefits and risks, how they differ from those who are responsible for the public health of the population,^50^ and how they vary across countries. In a study of public perceptions in the UK, a disease case caused by vaccination was weighted three-times as important as a disease case prevented by vaccination.^51^ This finding is consistent with high uptake of rotavirus vaccines in the UK (and other high-income settings) despite the clearly documented small elevated intussusception risk. However, perceptions around deaths are likely to be very different, and this issue is particularly important to understand in LMICs, where the overwhelming majority of rotavirus and intussusception deaths occur. Encouragingly, the initial safety studies from high-mortality settings have indicated no elevated risk of intussusception and coverage and uptake of rotavirus vaccines has been high. Good quality postlicensure surveillance will be essential to monitor the benefits and risks of rotavirus vaccination over time.

This analysis lends further support to the favourable benefit–risk profile of rotavirus vaccines in LMICs. Neonatal schedules have the potential to increase benefits further while reducing risks, but more efficacious rotavirus vaccines would be needed to achieve more substantial improvements in impact.

### Contributors

AC developed the model, did the analyses, produced the tables, and wrote the first draft of the manuscript. NH did a meta-analysis on the relative risk of intussusception in vaccine recipients. CS did a meta-analysis on the relative efficacy of a single dose of rotavirus vaccination. KVZ did the Bayesian analysis of intussusception risk by national under-5 mortality rate and produced the figure. All authors have read, contributed to, and approved the final version of the manuscript.

### Declaration of interests

BL reports personal fees from Takeda Pharmaceutical, Centers for Disease Control and Prevention Foundation and personal fees from Hall Booth Smith, outside the submitted work. All other authors declare no competing interests.
